# *Euphorbia characias*: Phytochemistry and Biological Activities

**DOI:** 10.3390/plants10071468

**Published:** 2021-07-17

**Authors:** Antonella Fais, Giovanna Lucia Delogu, Sonia Floris, Benedetta Era, Rosaria Medda, Francesca Pintus

**Affiliations:** Department of Life and Environmental Sciences, University of Cagliari, Monserrato, 09042 Cagliari, Italy; fais@unica.it (A.F.); delogug@unica.it (G.L.D.); s.floris@unica.it (S.F.); era@unica.it (B.E.); fpintus@unica.it (F.P.)

**Keywords:** *Euphorbia characias*, plant extracts, phytochemical constituents, biological activities

## Abstract

The aim of this review is to summarize all the compounds identified and characterized from *Euphorbia characias*, along with the biological activities reported for this plant. *Euphorbia* is one of the greatest genera in the spurge family of Euphorbiaceae and includes different kinds of plants characterized by the presence of milky latex. Among them, the species *Euphorbia characias* L. is an evergreen perennial shrub widely distributed in Mediterranean countries. *E. characias* latex and extracts from different parts of the plant have been extensively studied, leading to the identification of several chemical components such as terpenoids, sterol hydrocarbons, saturated and unsaturated fatty acids, cerebrosides and phenolic and carboxylic acids. The biological properties range between antioxidant activities, antimicrobial, antiviral and pesticidal activities, wound-healing properties, anti-aging and hypoglycemic properties and inhibitory activities toward target enzymes related to different diseases, such as cholinesterases and xanthine oxidase. The information available in this review allows us to consider the plant *E. characias* as a potential source of compounds for biomedical research.

## 1. Introduction

Euphorbiaceae is one of the largest flowering plant families, which is widely distributed in the world, especially in the tropical and temperate regions, and comprises about 300 genera and 8000 species [1]. The classification of Euphorbiaceae was revealed to be difficult due to the variability of habitat, morphology and genetics. This family showed a rich variety of chemical substances, especially rich in alkaloids and terpenoid compounds, and several potentially medicinal properties [2,3]. The genus *Euphorbia* comprises different kinds of plants, such as trees, lianas, herbs and shrubs. These plants are characterized by the presence of milky latex sap contained inside the laticifers, single specialized cells or articulated series of cells that permeate various tissues of the plant. Latex is a complex emulsion which consists of lipids, rubber, resin, starch and a variety of different proteins and enzymes. The physiological function of the latex is not completely known but it probably has a role as a water and nutrition reserve, and it seems to be involved in plant defense against phytopathogens and in sealing wounded areas [4].

**Figure 1:**
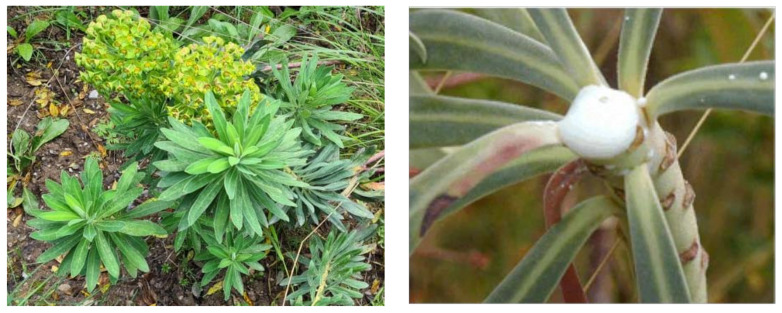
The Mediterranean shrub *E. characias.* Leaves and characteristic flowers are visible in the imagine on the left (**a**), while (**b**) clearly shows the milky latex that exudes from the cut branch of the plant (imagine modified from reference [4]).

Among Euphorbiaceae, the species *Euphorbia characias* L. is an evergreen perennial shrub up to about 1.5 m tall, with a bushy habit, widely distributed in Mediterranean countries. The inflorescence has a unique structure called the ‘cyathia’ which is arranged in rays growing from the bases of leaves. Leaves have a lanceolate structure 15 cm long, arranged along the stems. Latex permeates through all the plant and mainly exudes from the cut stems (Figure 1).

*E. characias* latex has been extensively studied and several proteins have been isolated and characterized. Among them, an enzyme named *Euphorbia* latex peroxidase (ELP) has been the object of numerous research papers due to its peculiar characteristics. This is a calmodulin-binding protein and its activity is therefore finely regulated by calcium ions. The presence of these ions, in addition to increasing the enzymatic activity, can even direct ELP towards different catalytic pathways using the same substrate [5]. A copper/TPQ-containing amine oxidase is also a part of the complex machinery of *E. characias* latex and the characterization of this enzyme made it possible to discover the key role of specific amino acids and domains in modulating substrate access into the active site of plant and mammalian amine oxidases [6]. Moreover, other enzymes have been purified and characterized and several nucleotide sequences are present in the GenBank database [7,8,9].

In recent years, scientists have made a great contribution to reporting the chemical constituents and biological properties of *E. characias*. However, there is no systematic review available that covers the important aspects of this plant, such as its non-protein composition. In order to provide new information for in-depth exploration and full use of this plant, we systematically and critically summarized the current findings on the non-protein compounds identified and characterized from *E. characias*. Furthermore, the biological activities of the isolated compounds or the tested extracts are also reported. The information available on this plant, reported in this review, allows us to explore their therapeutic potential, highlight gaps and provide the scientific basis for future study of this plant.

## 2. Chemical Constituents

Many chemical compounds with different structures have been identified and isolated from *E. characias*. In the following subsections, all these compounds, together with their nomenclature, are reported. The chemical structure of most of the compounds is reported to get an overview of the compounds with the structure immediately available, avoiding the well-known structure of compounds such as sugars, alkanes, alkenes or fatty acids. The reported molecules were identified from different parts of the plant (for example, in leaves, stems, latex, flowers or seeds) and the localization of each compound is also reported.

### 2.1. Terpenoids, Sterol Hydrocarbons, Fatty Acids and Cerebrosides

Terpenes are a class of hydrocarbon secondary metabolites, and their structure is built up from five-carbon isoprene units linked together. A rich diversity of structural classes of terpenes depend on different configuration degrees of unsaturation, oxidation, ring closures and functional groups. Terpenoids are primarily found in a wide variety of higher plants [10]. The terpenes and terpenoids are classified in hemiterpenes (1), monoterpenes (2), sesquiterpenes (3), diterpenes (4), sesterterpenes (5), triterpenes (6) and polyterpenes (many units), based on the number of isoprene units they present.

Polycyclic diterpenoids have been found in several plants of *Euphorbia* species and represent one of the major components of the lipid fraction [11]. In 2000, Appendino and colleagues identified 13 oxygenated diterpenoids from *E. characias* [12]. These compounds were isolated from an acetone extract from leaves and stems of the plant. Moreover, another two diterpenoid compounds were identified in the hexane extract of the latex. Table 1 shows the structure of these compounds which are abietane (compounds **1**–**6**), atisane (compounds **7**–**9**), kaurene (compound **10**)**,** pimarane (compounds **11**–**13**) and cembrane (compound **15**)-type diterpenoids. Diterpenes are important as natural products for potential applications as pharmacological agents in drug discovery due to their wide range of biological activities. In fact, antitumor, antimicrobial and anti-inflammatory activities are only some of the reported biological activities of this class of molecules [13]. Helioscopinolides A (**4**) and B (**5**) have displayed relevant activity against *Staphylococcus aureus* and a modest antibacterial property against *Moraxella catarrhalis* was previously found for helioscopinolide A [14]. Moreover, the *ent*-abietane compounds showed meaningful up-regulated expressions of matrix metalloproteinases [15], which not only have a role in the resolution phase of wound healing but also affect other responses linked to wound healing, such as re-epithelization and inflammation [16]. In a previous work [17], compound **10** (16*β*,17-dihydroxy-*ent*-kauran-3-one) was found to be cytotoxic at concentrations ranging from 2 to 50 µg/mL.

In 1983 and in 1984, Seip and Hecker isolated different lathyrane (**16a**–**f**) and jatrophane-type (**17a**–**h**) compounds from the acetone extract of *E. characias* latex [19,20], and the relative structures are reported in Table 2.

Lathyranes represent one of the main groups of tricyclic diterpenes with a 5, 11 and 3-membered ring system. These diterpenes may be considered derived from the hydrocarbon nucleus of casbane and its unsaturated analogue casbene. The fusion of rings A and B may have the configuration *trans* or *cis;* usually *trans* between rings A and B and *cis* between B and C. They may contain an epoxy function and double bonds.

Jatrophane diterpeses are macrocyclic compounds with a bicyclo[10.3.0]pentadecane skeleton, without the cyclopropane ring of lathyranes. Their structures vary for the configuration of the diterpene core, the number and position of the double bonds and the number of oxygen functions, which can be hydroxy, keto, epoxy, ether and ester groups. Natural jatrophane diterpenes, occurring exclusively in the Euphorbiaceae family, are in general polyacylated derivatives in which the acetyl, benzoyl, isobutanoyl, 2-methylbutanoyl or nicotinoyl are the acyl residues most frequently bounded.

Twelve new diterpenes were isolated from the whole plant. These compounds are based on a jatrophane skeleton and named euphocharacins a–l (**18a**–**l**) (Table 2) [21]. The biological activity of these compounds isolated from an ethyl acetate extract is reported as inhibition of the daunomycin-efflux activity of P-glycoprotein from cancer cells [22].

**Figure 2:**
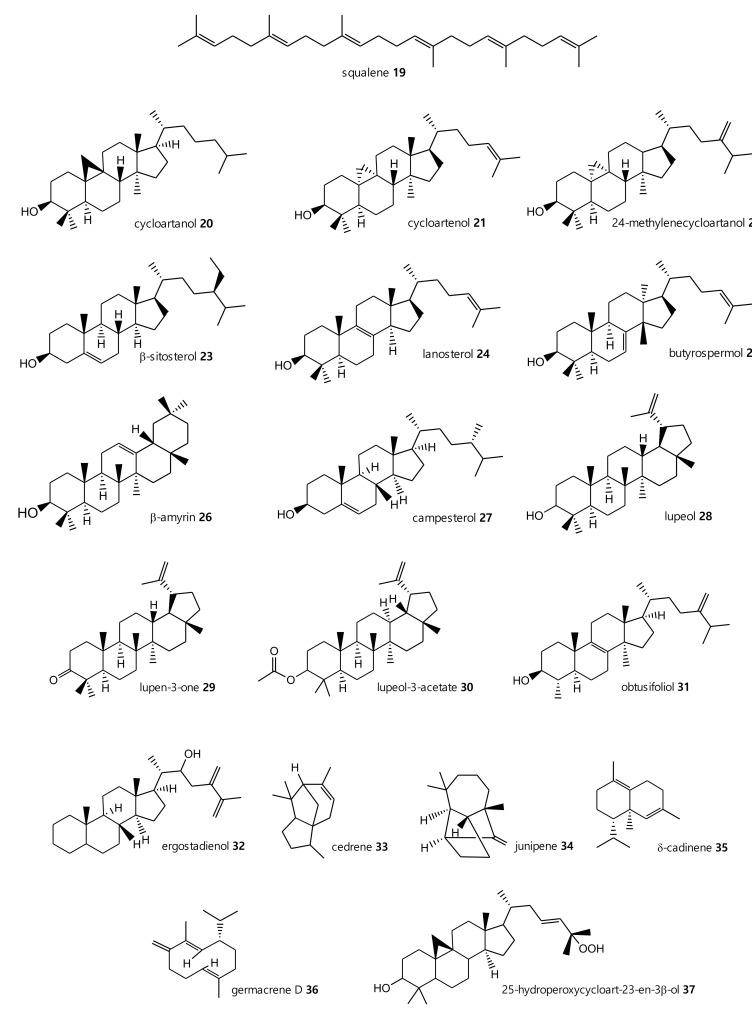
Triterpenes, sterols and sesquiterpenes identified from *E. characias* latex (compounds **19**, **20**, **22**, **24**, **25**, **28**, **31**–**36**) [18], seeds (compounds **20**, **22**–**24**, **26**, **27**) [24] and aerial parts (compounds **19**–**26**, **28**–**30**, **35**–**37**) [25,27].

Triterpenes are also predominant structures among the secondary metabolites identified in latex extract. Cycloartenol (**21**), 24-methylenecycloartenol (**22**), lanosterol (**24**) and lupeol (**28**) were found in significant amounts, but the major constituent was butyrospermol (**25**). Obtusifoliol (**31**), ergostadienol (**32**) and squalene (**19**) were present but in lower quantities. In the category of sesquiterpenes, cedrene (**33**), junipene (**34**), cadinene (**35**) and germacrene (**36**) were also identified [18]. Squalene and its derivatives, as well as other sterol compounds, were also identified in extracts from other sources, as reported in Figure 2.

In seeds of *E. characias*, the major lipids are neutral lipids, most of which are triacylglycerols, representing 80–97% of the total lipids [23]. The fatty acids identified in *E. characias* were myristic, palmitic, palmitoleic, stearic, oleic, vaccenic, linoleic, linolenic and arachidic acid (compounds **38**–**46**) [23,24,25,26]. Considering the literature, the published data about fatty acids composition present slightly differences, but it is clear that unsaturated fatty acids are more dominant than saturated in seeds [23,24]. Leaves showed a higher presence of linolenic acid (18:3) [23,25]. Moreover, oleic acid (18:1) is the major component of elaiosome and a role of this fatty acid in myrmecochory, by acting as a chemical cue for ants, has been proposed [26].

Cerebrosides represent a member of the sphingolipid class of lipids, which are also known to be distributed in Euphorbiaceae latex.

Nine cerebrosides were isolated and purified from the latex of *E. characias* (compounds **47**–**55**) (Table 3). Structurally, these cerebrosides are composed of a 1-*O*-*β*-d-glucopyranoside linked to a polyhydroxy sphingosine and a 2-hydroxy saturated or unsaturated fatty acid.

### 2.2. Phenolic and Carboxylic Acid Compounds

Phenolic and polyphenolic compounds are a large group of chemical substances with various chemical structures and activities. Chemically, they contain an aromatic ring with one or more hydroxyl groups, and they comprise simple molecules, such as phenolic acids, and more complex compounds, such as flavonoids. Phenolic and polyphenolic compounds are widely disseminated in the plant kingdom and constitute one of the most significant groups of secondary metabolites of plants, showing numerous bioactive properties, the most important being the antioxidant activity.

The chromatographic profile of a trichloroacetic acid (TCA) *E. characias* latex extract showed the presence of several carboxylic acids (aromatic and aliphatic) and alcoholic compounds [30]: benzoic acid (**56**), cinnamic acid (**57**), 4-hydroxybenzylalcohol (**58**), tyrosol (**59**), vanillic acid (**60**), *p*-coumaric acid (**61**), ferulic acid (**62**), sinapic acid (**63**), caffeic acid (**64**), 2-hydroxypropanoic acid (**65**), 2,3-dihydroxypropanoic acid (**66**), 3-hydroxypropanoic acid (**67**), 2-hydroxy-3-methylbutanoic acid (**68**), 4-hydroxybutanoic acid (**69**), 2-hydroxyesanoic acid (**70**), 3-phenylpropenoic acid (**71**) and 3-hydroxy-3-phenylpropenoic acid (**72**).

Furthermore, some flavonoids were identified in latex but especially in aerial parts of the plant. Flavonoids represent one of the major and ubiquitous pigments in plants. They are composed of two aromatic rings (A and B) linked by an oxygenated heterocycle (C).

**Figure 3:**
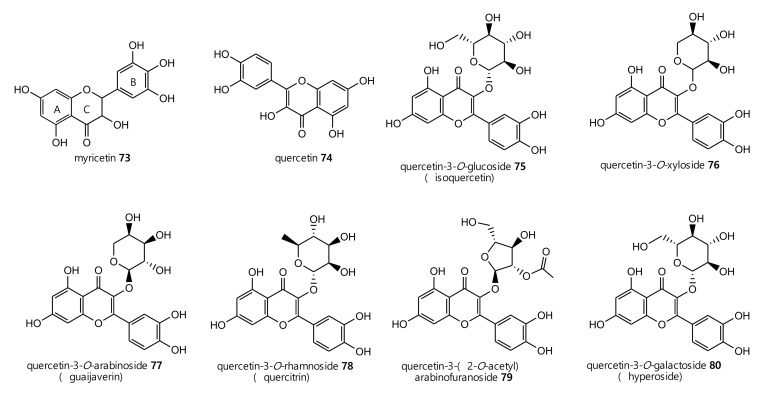

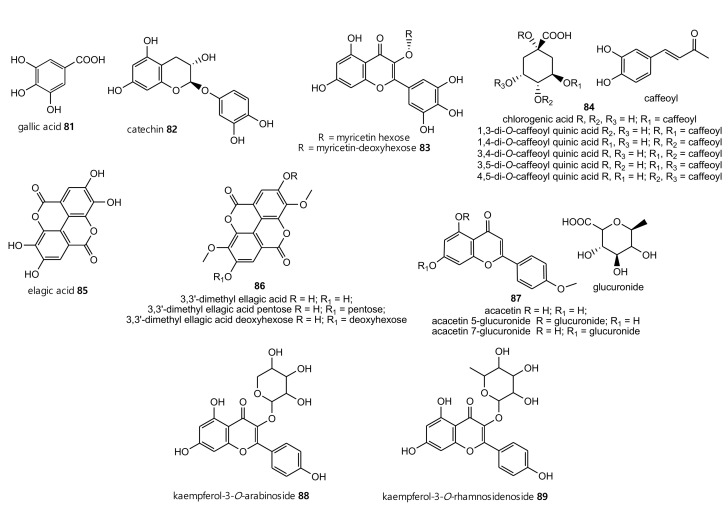
Phenolic compounds identified from *E. characias* latex (compounds **73**, **74**) [30] and aerial parts (compounds **74**–**89**) [25,31,32,33].

These secondary metabolites were mainly quercetin glycosides: quercetin-3-*O*-glucoside, quercetin-3-xyloside, quercetin-3-arabinoside, quercetin-3-rhamnoside (quercitrin), quercetin-3-(2-*O*-acetyl)arabinofuranoside, quercetin-3-*O*-galactoside and their precursor quercetin (**74**–**80**) (Figure 3). Other compounds, such as gallic acid, catechin, myricetin derivatives and ellagic acid derivatives, were identified in *E. characias* ethanolic extracts from leaves. Quercetin-3-(2-*O*-acetyl)arabinofuranoside (**79**) was the most abundant compound in ethanolic extract of both leaves and flowers, followed by quercetin-3-*O*-rhamnoside and quercetin-3-*O*-arabinoside (**78,77**) [31,32].

Wound-healing activity was reported for the methanolic extract of the aerial parts of *E. characias* subsp. *wulfenii* [33]. The combined activity of the constituents present in the extract, especially flavonoids such as guaijaverin **77**, quercitrin **78** and hyperoside **80,** seemed to be responsible of this effect. Biological activities of ethanolic extracts from leaves and flowers were also reported (see Section 3).

Finally, in the aerial parts of the plant, alkanes (**90**–**101**), alcohols (**102**–**107**), aldehydes (**108**–**113**), ketones (**114**, **115**) *α*-tocopherol (**116**), carboxylic acids (**117**, **118**) and sugars (**119**, **121**) were also identified (Table 4). Several of these metabolites change their concentrations in relation to the three phenological stages of the plant (preflowering, flowering and postflowering).

### 2.3. Natural Rubber and Rubber Particles

Natural rubber is an important polymer produced by numerous plants. The physiological role of rubber is not fully recognized, but it seems to be involved in disease resistance, wound healing or tolerance to environmental stress [35]. The main source of commercial natural rubber, at present, is the latex of *Hevea brasiliensis*, a member of the Euphorbiaceae family. This rubber has a characteristic high molecular weight (>1 million Da) and properties that are difficult to obtain with synthetic processes. Otherwise, the use of products (gloves or other medical devices) made from this natural rubber could cause an allergic reaction in sensitized persons. Two other plants seem to be a possible alternative to *H. brasiliensis*: *Parthenium argentatum* (Guayule) and *Taraxacum kok-saghyz*. However, new sources of natural rubber are needed [36].

A natural rubber from the latex of *E. characias* was identified, extracted and characterized [37]. This polymer was extracted by different methods, the better being extraction with acetic acid followed by treatment with cyclohexane and ethanol with a yield of 14.3% (*w*/*v*) of plant latex. It showed a low molecular weight of 93,000 Da and was revealed to have a *cis*-1,4-polyisoprene structure, typical of natural rubber. This low molecular weight rubber is different to that of *H. brasiliensis*, but it is similar to other Euphorbiaceae, such as *Euphorbia etherophylla* and *Euphorbia lactiflua* [38].

In latex-producing plants, the rubber biosynthesis occurs in the latex of laticifers, and rubber is compartmentalized in microscopic rubber particles constituted by a core of rubber encircled by a monolayer membrane and membrane-bound proteins [36]. Rubber particles of *E. characias* are spherical in shape with a diameter ranging from 0.02 to 1.2 mm, as observed by scanning electron microscopy [39]. The membrane-bound *cis*-prenyltransferase enzyme (“rubber transferase”, E.C. 2.5.1.20), which is the key enzyme in natural rubber biosynthesis, was identified in *E. characias* rubber particles, and the nucleotide and predicted amino acid sequence was determined [39].

## 3. Biological Activities

The biological activities of *E. characias* extracts are reported in the following subsections. Some extracts showed potential as sources of inhibitors of target enzymes related to different diseases, and the IC_50_ values are reported in Table 5. The activities of the compounds identified in the active extracts are also reported in Table 6.

### 3.1. Anti-Aging Properties

Tyrosinase, elastase and collagenase are some of the target enzymes in aging treatment or prevention. Tyrosinase (EC 1.14.18.1) is the key enzyme in the synthesis of melanin biopolymers. Melanin is produced in melanocytes by numerous enzymatic and non-enzymatic reactions. The first step is the conversion of tyrosine in 3,4-dihydroxyphenylalanine (l-DOPA) by a reaction of hydroxylation, followed by oxidation of l-DOPA to *o*-dopaquinone. These two reactions are catalyzed by tyrosinase and represent the rate-limiting step in melanin production. The subsequent steps involve oxidation and polymerization reactions and the action of other enzymes, such as the tyrosinase-related proteins. Melanin has an important skin photoprotective role, being able to absorb UV radiation and protect cells and tissues from the toxic effects of this radiation. However, inhibition of melanogenesis is particularly important on two fronts: cosmetics and medicine. An excess of melanin production can cause hyperpigmentation-related disorders such as melasma, age spots and freckles.

Extracts from *E. characias* aerial parts showed antimelanogenic properties. Among them, leaves ethanolic extracts showed the best activities (Table 5). Mushroom tyrosinase activity was inhibited with an IC_50_ of 34 µg/mL. The extract also showed antimelanogenic activity, inhibiting cellular tyrosinase activity by 47% and 57% at 50 and 100 µg/mL, respectively, using the B16F10 cellular model. A stronger effect than the standard compound, kojic acid, was determined [79]. As reported in Table 6, some of the compounds identified in flower and leaf ethanolic extracts of *E. characias* exhibited a well-documented inhibitory activity toward tyrosinase.

Quercitrin and two other quercetin derivatives were isolated from the methanolic extract of aerial parts of *E. characias* subsp. *wulfenii*, and these compounds were found to significantly inhibit elastase and collagenase activities [33]. Inhibition of these enzymes is linked with a wound-healing effect (see the next section “*In vivo wound-healing activity*”) and skin-aging prevention. Elastase (EC 3.4.21.36) and collagenase (EC3.4.24.3) are indeed enzymes that degrade the proteins elastin and collagen, involved in the elasticity and structure of the extracellular matrix. Thus, inhibition of these enzymes contributes to the prevention of wrinkle formation, characteristic of the skin-aging process.

### 3.2. In Vivo Wound-Healing Activity

In traditional medicine, *E. characias* latex and aerial parts are used to treat wounds. Significant wound-healing activity was reported from extracts of *E. characias* subsp. *wulfenii*. Active constituents were identified in methanolic extracts: quercetin-3-*O*-rhamnoside, quercetin-3-*O*-galactoside and quercetin-3-*O*-arabinoside. These compounds were present in extracts of the aerial parts of plant in different concentrations. These three phytoconstituents could be responsible for the wound-healing potential of the plant extract. Quercetin-3-*O*-rhamnoside was shown to be the most abundant compound in this extract of *E. characias* subsp. *wulfenii* [33]. Quercetin-3-*O*-rhamnoside and quercetin-3-*O*-arabinoside were also identified in alcoholic extracts of the leaves and stems of *E. characias* subsp. *characias*. In all extracts, quercetin-3-*O*-rhamnoside was the most abundant compound [25,31]. Bacterial infections or an over-production of reactive oxygen species (ROS) can reduce the process of wound healing. This process may be improved using antioxidant and antibacterial agents. The alcoholic extracts, fractions and isolated compounds from aerial parts of *E. characias* exhibited antimicrobial and antioxidant activities. Furthermore, the aerial parts of *E. characias* subsp. *wulfenii* possess anti-inflammatory activities [31,33]. Tsatsop Tsague et al. (2020) [80] reported the antioxidant and antimicrobial activities of quercetin-3-*O*-rhamnoside, one of the main compounds identified in aerial parts of *E. characias* species. Furthermore, quercetin-3-*O*-arabinoside was described to have antibacterial activity against *Bacillus cereus* and *Salmonella enteritidis* [81].

The quercetin glycosides mentioned above also showed collagenase and elastase enzyme inhibitory activity. In wound repair, collagenase and elastase activity catalyze the turn-over and restructuring of matrix components, which is a significant event in wound repair. These quercetin glycosides, with their activities, could contribute to the wound-healing activity shown by the extracts of the plant. All of this evidence supports the ethnomedicine use of the aerial parts of *E. characias* in folk medicine.

### 3.3. Cholinesterases Inhibition

Acetylcholinesterase (AChE, EC 3.1.1.7) and butyrylcholinesterase (BChE, EC 3.1.1.8) are enzymes associated with the pathology of Alzheimer’s disease (AD). In fact, one of the main causes linked to the onset of AD is the lack of acetylcholine. This neurotransmitter is hydrolyzed by both AChE and BChE, whose activities are enhanced in AD condition. Thus, the inhibition of these enzymes, enhancing the level of the neurotransmitter acetylcholine, represent one of the strategies in AD treatment.

Latex and aerial part extracts of *E. characias* were tested for their cholinesterase inhibition. Leaves, stems and flowers were extracted with water and ethanol and, as seen also from other enzyme inhibitions, the alcoholic extracts showed the best activity. The best result was obtained with leaves ethanolic extract on both cholinesterases, but it was more effective against BChE, even if the IC_50_ value was higher than the reference compound galantamine (Table 5). Latex extracted with trichloroacetic acid was tested only against AChE and showed good inhibition [30]. Analysis of latex composition showed the presence of several acid and phenolic compounds. Some of these compounds are reported to be AChE inhibitors, such as compounds **57** and **60**–**64** [82,83,84].

Table 6 reports some of the compounds identified in flower and leaf ethanolic extracts of *E. characias*, showing documented inhibitory activity against cholinesterase enzymes. The most present compounds in those extracts are the derivatives of quercetin and, among them, only the quercetin rhamnoside shows inhibitory activity against AChE (Table 6).

### 3.4. Xanthine Oxidase Inhibition

The purine metabolism in humans leads to the formation of uric acid. This final product, excreted in urine, is formed by the oxidation catalyzed by Xanthine oxidase (XO, EC 1.2.3.2) of hypoxanthine and xanthine, with concomitant production of ROS. The accumulation of uric acid results from an overproduction, due to an increased purine ingestion and metabolization, or from a decrease in its urinary excretion. Crystals of uric acid deposit in the joints, mostly in knee, elbow and ankle, causing severe pain and inflammation, and are responsible for the pathogenesis of gout. In order to control uric acid deposition, XO has been used as a therapeutic target, and its inhibitors are therefore used in the treatment of hyperuricemia and gout. Since XO is also a pro-oxidant enzyme, inhibitors with antioxidant activities are of much interest.

Inhibitory activity of leaves and flowers of *E. characias* was reported [32]. Only ethanolic extracts showed enzyme inhibition, which was higher than the standard inhibitor allopurinol (Table 5). Some of the compounds identified in flower and leaf ethanolic extracts of *E. characias* with a referred inhibitory activity against XO are reported in Table 6.

### 3.5. Hypoglycemic Properties

Hyperglycemia is a condition of an excessive amount of glucose in the blood serum, which may be associated with diabetes and related cardiovascular diseases. It requires emergency treatment. One of the main therapeutic strategies to treat diabetes is to decrease postprandial hyperglycemia by the inhibition of carbohydrate hydrolyzing enzymes, such as *α*-amylase and *α*-glucosidase. These enzymes break down complex carbohydrates and disaccharides to glucose, which is absorbed in the bloodstream. *α*-amylase (EC 3.2.1.1) is a key enzyme that catalyzes the endo-hydrolysis of *α*-d-(1, 4) glycosidic linkages in dietary starch, glycogen and carbohydrates with three or more glucose units. The oligosaccharides produced by *α*-amylase reaction are then degraded to free D-glucose by *α*-glucosidase (EC 3.2.1.3) in the small intestine. Thus, inhibition of these enzymes can suppress carbohydrate digestion, reduce glucose uptake and consequently, it may be helpful to reduce the postprandial glucose levels. Several enzyme inhibitors have been approved as antidiabetic drugs, such as acarbose and miglitol, but they induce side effects, and the identification of novel potential inhibitors is of great interest. Presently, only aerial parts of *E. characias* were tested for these activities and the results are encouraging for further investigation [32]. Both the enzymes are inhibited by plant extracts, but the best effect is showed against *α*-glucosidase. The *α*-glucosidase inhibitory activity of the ethanolic extracts was therefore found to be about 100 times higher than the standard compound acarbose (Table 6). Kinetic analysis showed that both extracts from leaves and flowers act as noncompetitive inhibitors against *α*-amylase, while mode of inhibition on *α*-glucosidase revealed that leaves and flowers act as noncompetitive and uncompetitive inhibitors, respectively. Moreover, it is noteworthy that the therapeutic drugs usually used for the treatment of hyperglycemia in diabetic patients, such as acarbose, show side effects probably correlated with a more significant inhibition of a-amylase if compared to α-glucosidase inhibition [85]. Leaf and flower ethanolic extracts showed a ratio between α-amylase and α-glucosidase inhibition higher than the ratio of acarbose, marking these extracts as good candidates for further study.

Table 6 reports the compounds identified in flower and leaf ethanolic extracts of *E. characias* showing documented inhibitory activity against *α*-amylase and *α*-glucosidase.

### 3.6. Antioxidant Activity

The biological activities highlighted for *Euphorbia* species included antioxidant activity. In these species, there is a vast number of different types of antioxidant compounds that could have a key role in preventing free radical chain reactions. Free radicals cause grave cell and tissue damage, which is the main cause of the aging process and the pathogenesis of several diseases.

The analysis of TCA extract of *E. characias* latex has highlighted high antioxidant activity; a higher amount of antioxidants was detected using ABTS^+•^ instead of DPPH^•^ assay [30]. Pintus et al. (2010) [4] reported, in *E. characias* latex, the presence of proteins that act as antioxidant enzymes. The antioxidant molecules and enzymatic proteins could act contemporarily as mechanisms of defense in the plant [30]. The activities of aqueous and alcoholic extracts from aerial parts of *E. characias* were evaluated with different assays. Leaf ethanolic extract exhibited significantly higher free radical scavenging activity if compared with aqueous extracts [31]. Moreover, leaf and flower ethanolic extracts showed no cytotoxic activity and inhibited H_2_O_2_-induced ROS generation in a cellular system [32]. Several compounds identified in *E. characias* are well-known antioxidant molecules [86,87,88,89,90,91,92,93,94,95,96,97,98,99,100,101,102,103,104,105,106,107] which could be responsible for the activity detected in the extracts.

### 3.7. Antiviral, Antimicrobial and Pesticidal Activities

The demand for bioactive compounds from natural sources is continuously increasing, because antibiotics and their extensive use led to the emergent problem of the multidrug resistance of microorganisms related to antibiotics and their extensive use. Several studies reported plant antimicrobials efficacy towards a great number of pathogens and food-borne agents that can cause viral infections and several diseases. In this context, aqueous and alcoholic extracts from aerial parts of *E. characias* have been investigated for their antiviral and antimicrobial efficacy. With regard to the antiviral effect, activities against the human immunodeficiency virus type 1 (HIV-1) reverse transcriptase-(RT-) associated RNA-dependent DNA polymerase (RDDP) and Ribonuclease H (RNase H) were evaluated. These activities are crucial for viral replication, and therefore they represent important drug targets for which there is a need for new drugs. All the extracts possessed anti-HIV activity and among them, ethanolic extracts were more active than aqueous extracts, and the ethanolic extract from flowers seemed to be the most effective inhibitor of HIV-1 RT-associated RNA-dependent DNA polymerase and Ribonuclease H. Among the compounds identified in flower and leaf ethanolic extracts of *E. characias,* compounds **74**, **78**, **81**, **84** and **89** showed documented HIV inhibitory activity [108,109,110,111].

Moreover, *E. characias* extracts were tested in order to evaluate an antagonistic activity against a panel of microorganisms: *Staphylococcus aureus*, *Bacillus cereus*, *Listeria monocytogenes*, *Escherichia coli*, *Salmonella typhimurium*, *Candida albicans*, *Saccharomyces cerevisiae*, *Aspergillus flavus* and *Penicillium chrysogenum*. Extracts from leaves appeared to possess the best antibacterial activity, followed by flower and stem extracts. Ethanolic extracts from leaves displayed the highest antibacterial activity towards all the tested Gram-positive bacteria: *B. cereus*, *L. monocytogenes* and *S. aureus* [31]. Among the compounds identified in this extract, antibacterial activity versus *S. aureus* was reported for compounds **75**, **77**, **81**, **82** and **84** [112,113,114,115,116]. Activity against *L. monocytogenes* and *B. cereus* was reported for compounds **77**, **81** and **82** [113,115,117] and **75** and **81** [112,114], respectively.

In addition to these properties, the antifungal activity of crude latex extract was reported [118]. The antifungal effect of plant latex seems to be related to the action of lysosomal hydrolases. These enzymes can be responsible for cell wall degradation, which results in a better entrance of antifungal drugs from extracellular medium. The concomitant use of latex and antifungal drugs may reduce the dose of drugs in the treatment of mycoses, therefore decreasing their side effects. *E. characias* latex showed an antifungal effect, alone and in combination with the synthetic imidazole drug, ketoconazole [118].

Plant chitinases exert a significant role in plant defense, acting on chitin-containing pathogens, and they also show antimicrobial, antiviral and insecticidal properties. In recent years, a chitinase was identified and purified from *E. characias* latex (ELC) [9]. ELC proved to be an effective management strategy against the insect *Drosophila suzukii*, which has recently caused important economic losses in Europe due to their attacks on developing soft fruits, by degrading the chitin exoskeleton of *D. suzukii*. Moreover, ELC at concentrations that were harmless to the host plants could be considered an environmentally friendly alternative to chemical pesticides, opening the door to develop sustainable agriculture [119].

## 4. Conclusions

Plant products are still major sources of innovative therapeutic agents, and since ancient times they have been widely used to treat many diseases, such as cancer, diabetes and cardiovascular and neurodegenerative diseases. Bioactive compounds from natural sources, as an alternative to synthetic molecules, are increasingly in demand, since they provide unlimited opportunities for new drug discoveries because of the unmatched availability of chemical diversity. In addition to the identification of new molecules with biological properties, there is growing evidence that known molecules are finding new applications through better understanding of molecular biology and clinical observations.

*E. characias*, cited in most ancient treatises of Greek and Latin medicine, is one of the oldest known medicinal plants of the Western tradition. The different phytochemicals, produced in the latex, seeds, steams, leaves and flowers of this plant, are terpenoids, sterol hydrocarbons, fatty acids, cerebrosides and phenolic and carboxylic acid compounds. In addition to these compounds, *E. characias* latex contains a low-molecular-weight natural rubber.

The *E. characias* biological properties reported in this review allow us to consider its wide potential therapeutic application. Indeed, it represents a source of inhibitors of target enzymes related to different oxidative stress-related diseases such as diabetes, Alzheimer’s and hyperpigmentation disorders, as well as microbial and viral infections. Once the bioactive compounds are identified, future prospects include further studies through in vitro and in vivo approaches in order to develop new, effective drugs. Moreover, vehiculation of active compounds represents an emerging technology and could be used to improve the delivery and efficacy of these compounds.

Although the plant *E. characias* needs additional physicochemical and chemical analyses, and a majority of its phytochemicals require further in-depth characterization for their therapeutic efficacy and safety, we believe that this review may contribute to provide the scientific basis for future study and full use of this plant.

## Figures and Tables

**Table 1:** Diterpenoids identified from aerial parts (leaves and stems) (compounds **1**–**10**, **12**–**14**) [12] and latex (compounds **11**,**15**) [18] of *E. characias*: structure of *ent-*abietanes, *ent*-atisanes, *ent-*kauranes, *ent*-pimaranes and cembrene.

Structure	Compound
***ent*-abietanes-1**	
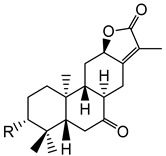	**1** 8*α*,14-dihydro-7-oxojolkinolide E R = H
	**2** caudicifolin R = OH (8*α*,14-dihydro-7-oxohelioscopinolide A)
***ent*-abietanes-2**	
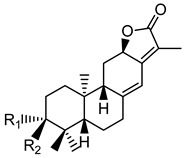	**3** jolkinolide E R_1_ = R_2_ = H (*ent*-abieta-8(14),13(15)-dien-16,12-olide)
	**4** helioscopinolide A R_1_ = OH; R_2_ = H
	**5** helioscopinolide B R_1_ = H; R_2_ = OH
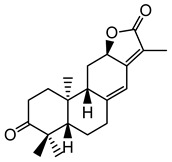	**6** helioscopinolide E
***ent*-atisanes**	
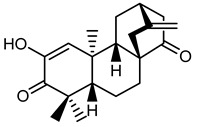	**7** *ent-*2-hydroxyatisa-1,16(17)-diene-3,14-dione
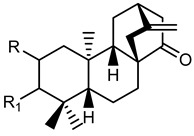	R R_1_ **8** *ent-*atis-16(17)-ene-3,14-dione H C=O **9** *ent-*3*α*-hydroxyatis-16(17)-ene-2,14-dione C=O OH
	***ent*-kauranes**
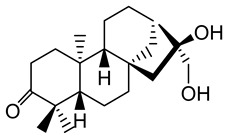	**10** 16*β*,17-dihydroxy-*ent-*kauran-3-one
	***ent*-pimaranes**
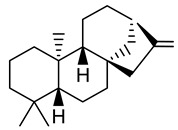	**11** kaurene
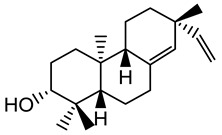	**12** *ent-*pimara-8(14),15-dien-3*α*-ol
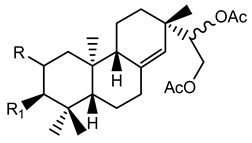	R R_1_ **13** 3*β*,15,16-triacetoxy-*ent*-pimar-8(14)-ene H OAc **14** 3*β*,15,16-triacetoxy-*ent*-pimar-8(14)-en-2-one C=O OAc
	**cembranes**
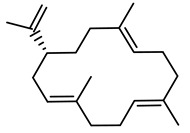	**15** cembrene

**Table 2:** Lathyranes and jatrophanes identified from *E. characias* latex (compounds **16a**–**f** and **17a**–**f**) [19,20], roots (compounds **17g**–**h**) and whole plant (compounds **18a**–**l**) [21].

Scheme	Compound
**lathyranes**	
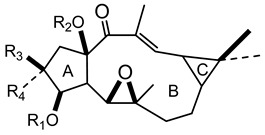	**16a** 15-*O*-acetyl-3-*O*-propionyljolkiol-5*β*,6*β*-oxide) R_1_ = COCH_2_Me; R_2_ = COMe; R_3_ = Me; R_4_ = H; **16b** 15-*O*-acetyl-3-*O*-*iso*-butyryljolkiol-5*β*,6*β*-oxide) R_1_ = COCHMe_2_; R_2_ = COMe; R_3_ = Me; R_4_ = H; **16c** 15-*O*-acetyl-3-*O*-tigloyljolkiol-5*β*,6*β*-oxide) R_1_ = COC(Me) ^E^CHMe; R_2_ = COMe; R_3_ = Me; R_4_ = H; **16d** 15-*O*-acetyl-3-*O*-benzoyljolkiol-5*β*,6*β*-oxide) R_1_ = COC_6_H_5_; R_2_ = COMe; R_3_ = Me; R_4_ = H; **16e** 15-*O*-acetyl-3-*O*-nicotinoyljolkiol-5*β*,6*β*-oxide) R_1_ = COC_5_H_4_N; R_2_ = COMe; R_3_ = Me; R_4_ = H; **16f** 2α-*O*-acetyl-3-*O*-*iso*-butyryl-15-*O*-nicotinoyljolkinol-5*β*,6*β*-oxide R_1_ = COCHMe_2_; R_2_ = COC_5_H_4_N; R_3_ = Me; R_4_ = OCOMe
**jatrophanes**	
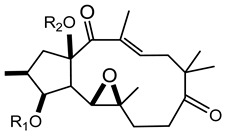	**17a** 15-*O*-acetyl-3-*O*-tigloylcharaciol-5*β*,6*β*-oxide) R_1_ = tigloyl (COC(Me) ^E^CHMe); R_2_ = COCH_3_; **17b** 15-*O*-acetyl-3-*O*-benzoylcharaciol-5*β*,6*β*-oxide) R_1_ = benzoyl (COC_6_H_5_); R_2_ = COCH_3_
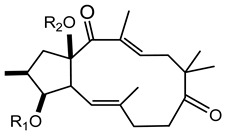	**17c** 15-*O*-acetyl-3-*O*-propionylcharaciol R_1_ = propionyl (COCH_2_Me); R_2_ = COCH_3_
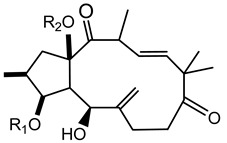	**17d** 15-*O*-acetyl-5*β*-hydroxyisocharaciol-3-*iso*-butyrate) R_1_ = *iso*-butyryl-(COCHMe_2_); R_2_ = COCH_3_; **17e** 15-*O*-acetyl-5*β*-hydroxyisocharaciol-3-tigliate R_1_ = tigloyl (COC(Me) ^E^CHMe*)*; R_2_ = COCH_3_; **17f** 15-*O*-acetyl-5*β*-hydroxyisocharaciol-3-benzoate R_1_ = benzoyl (COC_6_H_5_); R_2_ = COCH_3_
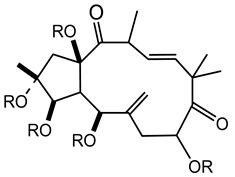	**17g** 2,5,8,15-*O*-triacetyl, nicotinoyl-2,5*β*,8-trihydroxyisocharaciol-3-benzoate R = acetyl (x 3), benzoyl, nicotinoyl; **17h** 2,5,8,15-*O*-triacetyl, nicotinoyl-2,5*β*,8-trihydroxyisocharaciol-3-tigliate R = acetyl (x 3), tigloyl, nicotinoyl;
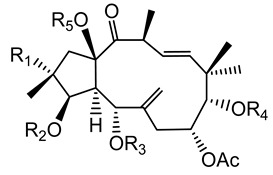	**18a**-l euphocharacin **18a** R_1_ = OH; R_2_ = Bz; R_3_ = Ac; R_4_ = Nic; R_5_ = Ac **18b** R_1_ = OH; R_2_ = Bz; R_3_ = Ac; R_4_ = Nic; R_5_ = H **18c** R_1_ = OH; R_2_ = Bz; R_3_ = Ac; R_4_ = Bz; R_5_ = H **18d** R_1_ = OH; R_2_ = MeBu; R_3_ = Ac; R_4_ = Nic; R_5_ = Ac **18e** R_1_ = H; R_2_ = Bz; R_3_ = Ac; R_4_ = Nic; R_5_ = H **18f** R_1_ = H; R_2_ = Bz; R_3_ = Ac; R_4_ = Nic; R_5_ = Ac **18g** R_1_ = H; R_2_ = *i*Bu; R_3_ = Ac; R_4_ = Nic; R_5_ = H **18h** R_1_ = H; R_2_ = *i*Bu; R_3_ = Ac; R_4_ = Nic; R_5_ = Ac **18i** R_1_ = H; R_2_ = Pr; R_3_ = Ac; R_4_ = Nic; R_5_ = Ac **18j** R_1_ = H; R_2_ = Ac; R_3_ = Ac; R_4_ = Nic; R_5_ = Ac **18k** R_1_ = H; R_2_ = *i*Bu; R_3_ = H; R_4_ = Nic; R_5_ = Ac **18l** R_1_ = OH; R_2_ = Bz; R_3_ = H; R_4_ = Nic; R_5_ = Ac Pr = propionyl; *i*Bu = isobutiryl; MeBu = 2-methylbutiryl; Bz = benzoyl; Nic = nicotinoyl

**Table 3:** Cerebrosides identified from the latex of *E. characias* (compounds **47**–**55**).

**cerebrosides 47–50** [28]
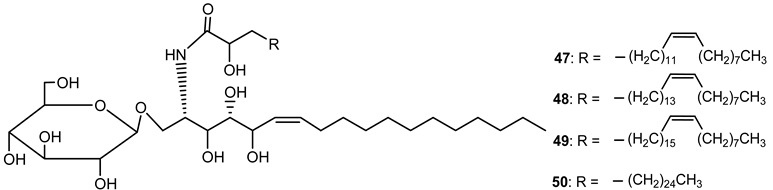
**47** (2S, 3S, 4R, 5R, 6Z)-l-*O*-(*β*-d-glucopyranosyl)-2-*N*-[2′R)-2′-hydroxy-(15′Z)-tetracosenoylamino]-6(Z)-octadecene-l,3,4,5-tetraol
**48** (2S, 3S, 4R, 5R, 6Z)-l-*O*-(*β*-d-glucopyranosyl)-2-*N*-[(2′R)-2′-hydroxy-(17′Z)-hexacosenoy1amino]-6(Z)-octadecene-l,3,4,5-tetraol
**49** (2S, 3S, 4R, 5R, 6Z)-l-*O*-(*β-*d-glucopyranosyl)-2-*N*-[(2′R)-2′-hydroxy-(19′Z)-octacosenoylamino]-6(Z)-octadecene-l,3,4,5-tetraol
**50** (2S, 3S, 4R, 5R, 6Z)-l-*O*-(*β*-d-glucopyranosyl)-2-*N*-[(2′R)-2′-hydroxyoctacosanoylamino]-(Z)-octadecene-l,3,4,5-tetraol
**cerebrosides 51–55** [29]
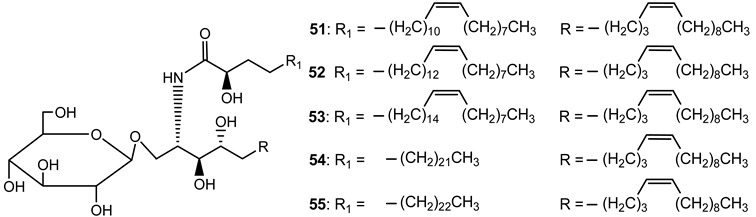
**51** (2S, 3S, 4R, 8Z)-l-*O*-*(β-*d-glucopyranosyl)-2-*N*-[(2′R)-2′-hydroxy-(15′Z)-tetracosenoylamino]-8(Z)-octadecene-1,3,4-triol
**52** (2S, 3S, 4R, 8Z)-l-*O*-(*β*-d-glucopyranosyl)-2-*N*-[(2′R)-2′-hydroxy-(17′Z)-hexacosenoyl]-8(Z)-octadecene-1,3,4-triol
**53** (2S, 3S, 4R, 8Z)-l-*O*-(*β*-d-glucopyranosyl)-2-*N*-[(2′R)-2′-hydroxy-(19′Z)-octacosenoylamino]-8(Z)-octadecene-l,3,4-triol
**54** (2S, 3S, 4R, 8Z)-l-*O*-(*β*-d-glucopyranosyl)-2-*N*-[(2′R)-2′-hydroxyhexacosanoylamino]-8(Z)-octadecene-1,3,4-triol
**55** (2S, 3S, 4R, 8Z)-l-*O*-(*β*-d-glucopyranosyl)-2-*N*-[(2′R)-2′-hydroxyheptacosanoylamino]-8(Z)-octadecene-l,3,4-triol

**Table 4:** Other compounds (**90**–**121**) identified from aerial parts of *E. characias*.

Compound	Part of the Plant	Reference
heptacosane **90**	Stems	[25]
nonacosane **91**	Leaves, Stems	[25]
pentacosene **92**		
hentriacontane **93**		
triacontane **94**		
tritriacontane **95**		
heneicosane **96**	Flowers	[34]
docosane **97**		
tricosane **98**		
pentacosane **99**		
heptacosane **100**		
nonacosane **101**		
phytol **102**	Leaves, Flowers	[25,34]
tetracosanol **103**	Stems	[25]
hexacosanol **104**	Leaves, Stems	[25]
octacosanol **105**		
inositol **106**		
myo-inositol **107**		
octacosanal **108**		
nonanal **109**	Flowers	[34]
tridecanal **110**		
tetradecanal **111**		
hexadecanal **112**		
hexacosanal **113**		
6,10,14-trimethyl-2-pentadecanone **114**		
farnesyl acetone **115**		
*α*-tocopherol **116**	Leaves	[25]
hexadecanoic acid **117**	Flowers	[34]
pyroglutamic acid **118**	Leaves, Stems	[25]
D-glucose **119**		
D-fructose **120**		
sucrose **121**		

**Table 5:** Inhibitory activities of *E. characias* extracts. The inhibitory activity is expressed as IC_50_ values. Kojic acid, galantamine, allopurinol and acarbose are reported as standard inhibitors. The mode of inhibition, when known, is shown in square brackets.

Part of the Plant/Reference Compounds	Extract	IC_50_ (µg/mL)					
		Tyrosinase	AChE	BChE	XO	*α*-Amylase	*α*-Glucosidase
Leaves	Water	120 ± 10 [mixed-type]	(4.2 ± 0.25) × 10^3^	NI	>200	74.02 ± 3.06	1.4 ± 0.11
	Ethanol	34 ± 2 [competitive]	600 ± 56	390 ± 40	68.9 ± 6.6 [mixed-type]	25.41 ± 1.42 [noncompetitive]	0.8 ± 0.03 [noncompetitive]
Stems	Water	(1.80 ± 0.13) × 10^3^	(6.9 ± 0.71) × 10^3^	NI	-	-	-
	Ethanol	(1.10 ± 0.090) × 10^3^	(5.8 ± 0.43) × 10^3^	NI	-	-	-
Flowers	Water	490 ± 25	(5.25 ± 0.35) × 10^3^	(4.20 ± 0.39) × 10^3^	>200	109.12 ± 10.36	1.1 ± 0.07
	Ethanol	150 ± 11	600 ± 56	(1.22 ± 0.08) × 10^3^	85.5 ± 6.4 [mixed-type]	29.39 ± 1.41 [noncompetitive]	0.9 ± 0.04 [uncompetitive]
Kojic acid		0.8 ± 0.03	-	-	-	-	-
Galantamine		-	0.27 ± 0.07	8.12 ± 0.61	-	-	-
Allopurinol		-	-	-	0.012	-	-
Acarbose		-	-	-	-	8.04 ± 0.65	90 ± 7.3

NI: no inhibition.

**Table 6:** Compounds identified in *E. characias* flower and leaf ethanolic extracts with referred inhibitory activity.

Compound	Tyrosinase	AChE	BChE	XO	*α*-Amylase	*α*-Glucosidase
quercetin **74**	[40,41,42]	[43,44,45,46]	[43,44,46,47]	[48,49]	[50,51]	[50,52,53,54]
quercetin-3-*O*-glucoside **75**	[41,55,56]	-	-	[57]	[58]	[59]
quercetin-3-*O*-xyloside **76**	[60]	-	-	-	-	-
quercetin-3-*O*-arabinoside **77**	[60]	-	-	-	-	[61]
quercetin-3-*O*-rhamnoside **78**	[62]	[63]	-	-	-	[52]
gallic acid **81**	[64,65]	[46]	[46]	[66]	NI [67]	[67]
catechin **82**	[68]	[69]	[69]	[70]	[71]	[72]
di-*O*-caffeoylquinic acid **84**	[73,74]	-	-	[75]	[76]	[77]
kaempferol-3-*O-*arabinoside **88**	-	-	-	-	-	[61]
kaempferol-3-*O-*rhamnoside **89**	[78]	[63]	-	-	-	[61]

NI = no inhibition.

## Data Availability

Data is contained within the article.
